# 

**Published:** 1881-01

**Authors:** 


					﻿SUR^GENLB^GFWv
Voi. XVI.
Janùarv. 1881
.No. 4.
IH
ISTOURY
m É é Oí# MB
e .P IT Q R
elmirany
				

